# Engineering Large Porous Mannitol-PVA Microparticles for Extended Drug Delivery via Spray Drying

**DOI:** 10.3390/pharmaceutics17091135

**Published:** 2025-08-30

**Authors:** Karnkamol Trisopon, Ornanong Suwannapakul Kittipongpatana, Neungreuthai Chomchoei, Nara Yaowiwat, Phennapha Saokham

**Affiliations:** 1 Department of Pharmaceutical Sciences, Faculty of Pharmacy, Chiang Mai University, Chiang Mai 50200, Thailand; karnkamol.t@cmu.ac.th (K.T.); ornanong.sk@gmail.com (O.S.K.); 2 Faculty of Pharmacy, Institute of Entrepreneurial Science Ayothaya, Phra Nakhon Si Ayutthaya 13000, Thailand; neungreuthai.c@iesa.ac.th; 3 School of Cosmetic Science, Mae Fah Luang University, Chiang Rai 57100, Thailand; nara.nan@mfu.ac.th; 4 Green Cosmetic Technology Research Group, School of Cosmetic Science, Mae Fah Luang University, Chiang Rai 57100, Thailand

**Keywords:** large porous particles, PVA, mannitol, diclofenac sodium, spray drying, extended drug delivery

## Abstract

**Background:** Large porous particles (LPPs) offer significant potential in drug delivery due to their porous structure and suitable particle size and shape, which can improve powder dispersibility and control drug release. **Methods:** In this study, sustained-release large porous microparticles of mannitol, PVA, and diclofenac sodium (MPDs) were developed using a spray drying technique. The influence of PVA co-spray drying and its concentration (0–40%) on the characteristics of the spray-dried particles was investigated. **Results**: Co-spray drying with PVA enhanced particle morphology, producing MPDs with a spherical shape and smooth surface, which minimized particle adhesion. This improvement correlated with a low Carr’s Index value (17.56%), indicating favorable particle dispersibility and aerosol performance. The large geometric diameter (>5 μm) of the MPDs, coupled with their low bulk density (<0.1 g/cm^3^), suggested potential for inhalation use. FTIR, XRD, and DSC analyses revealed that PVA altered the polymorphic form of mannitol, with the MPDs exhibiting a mixture of the α and δ forms. In vitro dissolution tests demonstrated that PVA co-spray drying effectively prolonged drug release, with the formulation containing 40% PVA (MPD-4) showing an optimal release profile. The release kinetics followed first-order Higuchi models, suggesting drug release occurred through a matrix diffusion mechanism facilitated by the porous structure. **Conclusions**: These findings demonstrate the feasibility of engineering large porous microparticles with tailored release characteristics and physicochemical properties suitable for further development in inhalable or other controlled-release dosage forms.

## 1. Introduction

Large porous particles (LPPs) have emerged as a versatile platform in modern drug delivery systems due to their unique structural characteristics, including low density and large geometric size. These attributes enable improved powder dispersibility, reduced particle–particle cohesion, and enhanced aerodynamic performance. In pulmonary applications, LPPs have gained particular attention for use in dry powder inhalers (DPIs), which are established delivery systems valued for their portability, stability, and ease of use [1]. However, conventional carrier-based DPIs rely on drug detachment from large excipient particles and are limited by restricted drug loading, often resulting in inconsistent delivery to the lungs [2].

LPP technology has been explored as an alternative to address these limitations. LPPs are defined as particles with a low-density structure (bulk density < 0.1 g/cm^3^) and a large geometric particle size (5–30 μm) [3]. These characteristics can also yield calculated aerodynamic diameters that may be suitable for inhalation, potentially improving deposition in the lower respiratory tract [4]. These particle characteristics provide a suitable aerodynamic diameter (<5 μm), a range considered favorable for aerosolization performance [5]. Furthermore, the porous structure of LPP allows for the incorporation of functional excipients and active pharmaceutical ingredients (APIs), providing opportunities to engineer drug release profiles tailored to specific therapeutic needs.

Spray drying is a versatile and widely used technique for producing LPPs, and is a single-step process in the conversion of liquid feed into dry particles. This technique also serves as an effective co-processing technique for producing advanced pharmaceutical particles by integrating APIs and excipients to form particles with tailored characteristics such as size, shape, porosity, and surface morphology. By using the co-spraying technique, the advantages of excipients are combined and undesirable properties are reduced, leading to improved material functionality [6]. In our previous work, LPP particles were developed by co-spray drying mannitol with ammonium bicarbonate (poring agent). This LPP, designed for immediate-release use, showed favorable morphology, particle size, and density characteristics for powder inhalation [7]. There are concerns regarding rapid-release formulations, where the drug concentration peaks quickly and then declines rapidly, which can lead to undesirable side effects initially and insufficient therapeutic effects later, particularly for drugs whose efficacy depends on maintaining specific tissue or blood concentrations [8].

On the other hand, extended-release formulations are designed to retain the drug in the target site for an extended period and gradually release it locally at therapeutic levels. Thus, extended-release formulations offer several advantages over traditional formulations, as they serve to maintain consistent therapeutic drug levels, enhancing local efficacy and reducing systemic side effects [9]. Various synthetic polymers have been studied as drug carriers for DPIs, including polyvinyl alcohol (PVA), which is biologically inert and can prolong drug release. Co-spray drying of PVA has been reported to improve the morphology of microparticles, producing spherical shapes with smooth surfaces and a low tendency for particle shrinkage [10]. Previous work conducted on the composite of ciprofloxacin-loaded PVA particles revealed that high PVA incorporation could extend drug release over 24 h [11].

Mannitol is a sugar alcohol that has been widely utilized as a drug carrier in DPI formulations. It is highly stable, crystalline, non-hygroscopic, and biocompatible, making it ideal for pulmonary applications [12]. Mannitol is also stable at high temperature, which facilitates modifications through spray drying. Spray-dried mannitol was investigated as a drug carrier for inhalation, where the surface morphology was primarily influenced by the outlet temperature, which affected aerodynamic performance [13,14]. In addition, Peng et al. developed nanoporous mannitol as a drug carrier for pulmonary delivery using spray drying. Their study revealed that porous spray-dried mannitol exhibited superior deposition efficiency compared to non-porous particles [15].

Diclofenac sodium is a non-steroidal anti-inflammatory drug (NSAID) known for its potent anti-inflammatory, analgesic, and antipyretic properties. It works by inhibiting cyclooxygenase (COX) enzymes, primarily COX-2, reducing the synthesis of prostaglandins that mediate inflammation, pain, and fever. However, its non-selective inhibition of COX enzymes can lead to potential side effects such as gastric irritation, ulceration, and bleeding, especially when administered orally. For these reasons, exploring new delivery routes such as pulmonary drug delivery has gained attention as a strategy to reduce side effects and avoid first-pass metabolism [16]. In addition, a sustained-release inhalable formulation could maintain therapeutic drug concentrations for extended periods, thereby reducing dosing frequency, an advantage particularly relevant for short half-life drugs such as diclofenac sodium, and improving patient adherence. Moreover, sustained release may attenuate peak–trough fluctuations in drug concentration, potentially lowering the risk of local side effects, similar to the benefits reported for other inhaled sustained release therapies [17].

This study focuses on the formulation and physicochemical characterization of large porous spray-dried PVA-mannitol microparticles designed for extended-release formulations. To achieve this, mannitol was co-spray dried with PVA, and diclofenac sodium was a model drug. Ammonium bicarbonate was incorporated as a porogen to create a porous structure via thermal decomposition during spray drying. The effect of PVA co-spraying and its concentration on the porous architecture, particle morphology, physicochemical properties, and polymorphic behavior of the spray-dried particles was systematically investigated. Furthermore, an in vitro dissolution study was performed to evaluate drug release kinetics and evaluated the role of PVA in modulating the porous structure and controlling the release mechanism.

## 2. Materials and Methods

### 2.1. Materials

Mannitol (CAS No. 1344-09-8; Product code: 2305169781) was purchased from Kamaus (Cherrybrook, NSW, Australia), while polyvinyl alcohol (PVA; Mw 85,000–124,000; CAS No. 9002-89-5; product code: 1003548177) was purchased from Sigma-Aldrich (Burlington, MA, USA). The ammonium bicarbonate (AB) (CAS No. 1066-33-7; Product code: 0122000500) was supplied by Loba Chemie (Wodehouse Road, Colaba, Mumbai, India). Diclofenac sodium was a gift from the Bangkok Lab and Cosmetics Public Company Limited (Ratchaburi, Thailand).

### 2.2. Spray Drying of Large Porous Microparticles of PVA-Mannitol-Diclofenac Sodium (MPDs)

The preparation of MPDs was adjusted from the method described in our previous work [7]. The feedstock solution for spray drying was prepared with 2.5% *w/v* total solid content, and the effect of varying PVA concentrations was investigated. Various PVA concentrations (10–40% *w/w*) were dispersed in distilled water and heated to 80 °C while continuously stirring until it dissolved completely. After cooling down the PVA solution to room temperature, mannitol, AB (30% of dry sample weight), and diclofenac sodium were added into the solution and stirred to obtain a clear solution. The large porous microparticles were produced using a B-290 Mini Spray Dryer (Buchi, Flawil, Switzerland), where the inlet temperature, outlet temperature, aspirator setting, and pump rate were controlled at 130–135 °C, 65 °C, 75%, and 10–20%, respectively. The spray-dried mannitol with poring agent (MA) and mannitol co-spray-dried with diclofenac sodium (MD) were prepared as control samples.

### 2.3. Scanning Electron Microscope (SEM)

The morphology of the large porous microparticles was examined with a CLARA field emission scanning electron microscope (FESEM) from TESCAN (Brno, Czech Republic). Prior to analysis, the samples were dried at 60 °C and observed under low vacuum conditions (0.7 to 0.8 Torr) with an acceleration voltage of 10 to 20 kV.

### 2.4. Particle Size Analysis

The particle size and size distribution were measured using a laser diffraction technique with a Mastersizer 3000 (Malvern Instrument, Worcestershire, UK). The sample powder was dispersed in isopropanol as a tested medium (refractive index of 1.38) and measured at room temperature, while D_10_, D_50_, D_90_, average geometric diameter (μm), and span were recorded. The test was repeated at least three times. Then, the aerodynamic diameter (D_aer_) was calculated using Equation (1) [18].

(1) $$D_{aer}=D_{mean}(\sqrt{\rho_{t}/\rho_{0}x})$$

where D_mean_ is the particle mean diameter (μm) according to laser diffraction results. $\rho_{t}$ is the powder tapped density, while $\rho_{0}$ is a standard reference density of a spherical particle (1 g/cm^3^). The shape factor (x) was assigned based on particle morphology observed in SEM images and literature values reported by Hassan et al. [19]. Samples with smooth spherical morphology were assigned x = 1.0, particles with rough/pollen-like spherical morphology were assigned x = 1.2, and particles with irregular plate-like morphology were assigned x = 1.5.

### 2.5. Specific Surface Area (SSA)

The sample surface area was measured using a BET surface analyzer (Nova2200e, Quantachrome, Boynton Beach, FL, USA). Prior to analysis, the samples were degassed under vacuum at 120 °C for 6 h, where SSA was determined using the multi-point Brunauer–Emmett–Teller (BET) equation.

### 2.6. Powder Density and Powder Flowability

Powder density including bulk, tapped, and true densities were investigated. Bulk density ($\rho_{b}$) and tapped density ($\rho_{t}$) were measured using a graduated cylindrical method. The sample powder was weighed and gently poured into a 25 mL graduated cylinder with 1 mL readable, where the appearance volume was read and recorded as a bulk volume. After that, the sample was continuously tapped 1250 times using a Jolting volumeter (Stav 2003, Erweka, Langen, Germany). The resulting volume after tapping was read and recorded as tapped volume. Powder true density ($\rho_{\mathrm{true}}$) was evaluated using an Accupyc II 1340 pycnometer (Micromeritics, Norcross, GA, USA). The test was conducted using a gas displacement method with helium gas at 25–30 °C and repeated ten times. Then, powder porosity (%) was calculated using Equation (2).

(2) $$\mathrm{Porosity}\;(\%)=(1-(\rho_{b}/\rho_{t}))\times 100$$

Powder flowability was evaluated using Carr’s index (%), which determined the powder cohesiveness. Carr’s index values were calculated using Equation (3).

(3) $$\mathrm{Carr}’s\;\mathrm{index}\;(\%)=[(\rho_{t}-\rho_{b})/\rho_{t}]\times 100$$

### 2.7. FT-IR Spectroscopy

The FT-IR spectra were obtained using an Invenio R FT-IR spectrophotometer (Bruker, Billerica, MA, USA). The sample powders were homogeneously blended with KBr and analyzed with attenuated total reflectance (ATR) mode. The FT-IR spectra of mannitol, diclofenac sodium, PVA, and representative MPDs were acquired.

### 2.8. X-Ray Diffraction (XRD)

The XRD patterns of the samples were analyzed using a Miniflex II (Rigaku, Tokyo, Japan) in reflection mode. The diffractogram was registered at a Bragg angle (2θ) range of 10–60° with a scanning rate of 2.5 per min.

### 2.9. Differential Scanning Calorimetry (DSC)

The thermal properties of the samples were obtained using a DSC-1 instrument (Mettler-Toledo, Greifensee, Switzerland). The samples were analyzed over a temperature range of 25–300 °C with 10 °C/min of heating rate. The thermal transition parameters, including onset temperature (T_o_), peak temperature (T_p_), end temperature (T_e_), and peak area, and enthalpy change (ΔH) were evaluated.

### 2.10. In Vitro Drug Release Testing and Drug Content Analysis

The in vitro drug release testing was evaluated using a dialysis bag diffusion method with a dialysis tube of 10K molecular weight cut-off and 35 mm internal diameter (Thermo Scientific, Waltham, MA, USA). The sample powder (500 mg, which was equivalent to 50 mg of diclofenac sodium) was accurately weighed and loaded in a dialysis tube and sealed. The dialysis bag was submerged in a 250 mL of phosphate buffer, pH 7.4 (dissolution medium), and the temperature was maintained at 37 °C throughout the study. The test was conducted using a paddle apparatus with 50 rpm of paddle speed, while 1 mL of the samples were withdrawn from the vessel at 1, 2, 4, 6, 8, 12, and 24 h and replaced with fresh medium for each sampling [20]. The concentrations of diclofenac sodium were analyzed using high-pressure liquid chromatography (HPLC) on an Agilent 1260 Infinity Quaternary LC (Agilent Technologies Inc., Santa Clara, CA, USA). The analysis was conducted using an Alltech Apollo C18 5u column (150 mm × 4.6 mm), while the flow rate was set at 0.3 mL/min with injection volume 1 μL and 276 nm of detection wavelength. The cumulative drug release (%) was calculated, while the test was conducted in triplicate. The drug release kinetics of the co-spray-dried particles were evaluated with various kinetic models including zero-order release, first-order release, Higuchi, Hixson-Crowell, and Korsmeyer-Peppas models. The correlation coefficient (r^2^) was calculated to explain the release mechanism. Additionally, the residual sum of squares (RSS) was computed to quantify the difference between the experimental and predicted drug release values, as described by Equation (4).

(4) $$\mathrm{RSS}=\sum_{i=1}^{n}{\left(Q_{t,i}^{\mathrm{observed}}-\;Q_{t,i}^{\mathrm{predicted}}\right)}^{2}$$

where *n* denotes the number of data points used for model fitting, while *Q*_*observed*_ and *Q*_*predicted*_ represent the experimentally measured and model-predicted cumulative drug release at each time point, respectively.

The percentage of drug content was determined by dissolving sample powder in phosphate buffer pH 7.4 and diluted to a suitable concentration. The solution was filtered with a syringe filter 0.45 μm before analysis. The amount of diclofenac sodium entrapped in a sample was measured using an HPLC analysis as described above. The percentage of drug content was calculated, and the test was carried out in triplicate.

### 2.11. Statistical Analysis

The experiments were conducted at least in triplicate and are presented as average ± standard deviation (SD). Significant differences were analyzed using one-way analysis of variance (ANOVA) followed by Tukey’s Honestly Significant Difference (HSD) test at a 95% confidence level (*p* < 0.05) using SPSS software (Version 19.0).

## 3. Results and Discussion

### 3.1. Scanning Electron Microscope (SEM)

**Figure 1:**
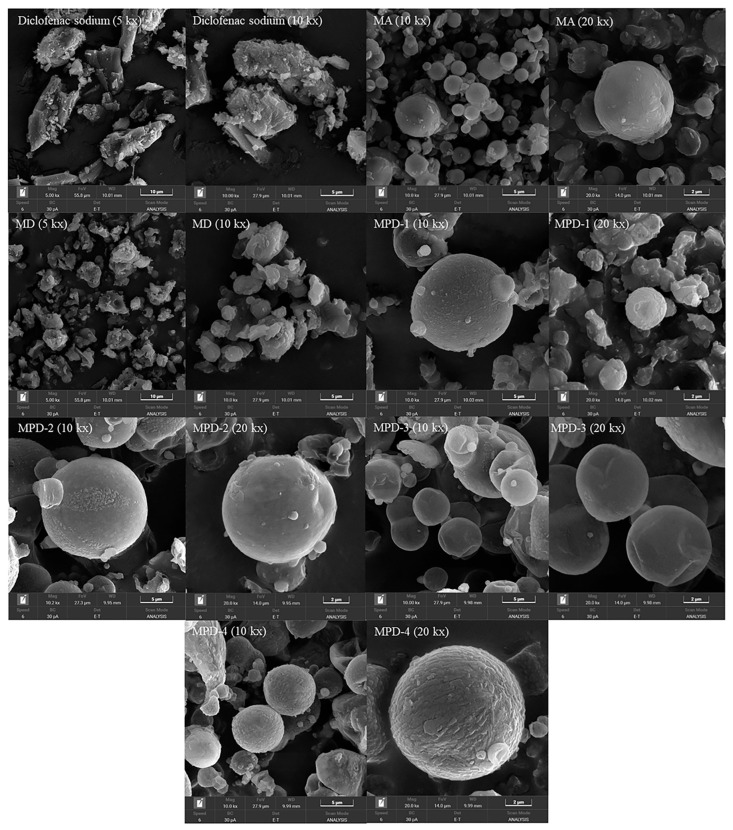
SEM images of diclofenac sodium, MA, MD, and MPDs.

The morphological characteristics of MPDs and control samples are shown in Figure 1. Before spray drying, diclofenac sodium exhibited crystal particles with an irregular shape. The spray-dried mannitol with a poring agent (MA) formed spherical particles with smooth surfaces, with particle sizes ranging from 2 to 5 μm. Mannitol co-spray dried with diclofenac sodium showed particles with more irregular shape and surface roughness, as well as some particle aggregation. The incorporation of diclofenac sodium may affect the diffusion rate of the solute, lowering the evaporation rate, leading to the formation of uneven, irregular particles as suggested in earlier studies [21]. This effect was also observed in the MPD particles with low PVA concentration (MPD-1). However, the PVA co-spray drying improved the morphology of MPD particles, where the increase in PVA concentrations provided the particles with a more spherical shape and smoother surface. The addition of PVA increased the solution viscosity, which slowed down the rate of diffusion motion, resulting in a more uniform distribution of the solute molecules. Particles with smoother surfaces and increased size were observed in formulations with higher solution viscosity. This may be due to the enhanced droplet strength and more rigid shell formation during spray drying. Similar effects have been reported in previous studies [10,22]. The smooth surface was significantly beneficial for aerodynamic performance, as this characteristic provided lower contact points thus reducing adhesion force between the particles [23]. Moreover, the highest used PVA concentrations (40% *w/w*) provided the particles with the most roughness surface among the MPDs, suggesting that the solution viscosity was too high, leading to uneven droplet formation.

### 3.2. Particle Size Analysis

The particle size and aerodynamic diameter results are presented in Table 1. The MD particles exhibited the smallest average particle size among all samples. The MA formulation, which was co-spray dried with a poring agent, exhibited an increased average particle size but a slightly reduced aerodynamic diameter. As ammonium bicarbonate evaporated during the spray drying process, it caused the expansion of the particle structure while creating porosity in the structure [15]. The MPD particles, produced by co-spray drying with PVA, exhibited a progressive increase in average particle size (13.74–18.38 μm) with increasing PVA concentrations. The addition of PVA resulted in the formation of larger particles with reduced shrinkage. This effect may be attributed to increased solution viscosity and the enhanced rigidity of the particle shell during spray drying, as supported by previous studies [24], The aerodynamic diameter was estimated based on the average particle size (geometric diameter), powder density, and shape factor. Porous particles are generally characterized by a tapped density of less than 0.4 g/cm^3^ and an aerodynamic diameter below 5 μm [18]. The shape factor value was considered based on sample morphology in the SEM image. MA, MPD-2, MPD-3, and MPD-4 were considered spherical particles, while MD and MPD-1 were considered plate-shaped and pollen shape II according to the literature values reported by Hassan et al. [19]. The results revealed that MPD-2 to MPD-4 had an aerodynamic diameter in the range of 1–5 μm, while their geometric diameter was larger than 5 μm. These results suggest the potential of the particles for inhalation drug delivery based on their physical characteristics. The combination of a relatively large geometric diameter, which can enhance powder dispersibility, and a low aerodynamic diameter, which is generally considered favorable for respiratory tract deposition, is consistent with design principles used in the development of inhalation formulations [3].

### 3.3. Specific Surface Area (SSA)

The SSA of the particles was primarily influenced by particle morphology and density. The presence of ammonium bicarbonate (AB) contributed to the formation of porous structures, as reflected by the increased surface area of the particles. This effect is consistent with previous reports on the use of AB as a poring agent in spray-dried systems [25]. In this study, the concentration of the poring agent was consistent across all samples. The MA particles showed an SSA value of 4.356 m^2^/g, while the co-spray drying of mannitol with diclofenac sodium moderately decreased the SSA value of the MD particles (Table 2). This combination provided some particle agglomeration, resulting in a lower SSA. On the other hand, co-spray drying with PVA significantly impacted the particle surface area. The SSA values of MPDs gradually increased with PVA concentrations up to 30% of PVA (MPD-3). However, at 40% PVA (MPD-4), the SSA decreased to the lowest value. These results suggest that PVA contributed to pore formation in the MPD particles, as it improved the strengthening of the droplet and reduced the tendency of particle shrinkage during spray drying. The increase in SSA values may be attributed to the formation of porous structures, which occur as solvent evaporates and leaves void spaces within the particles, which has been described in previous studies [10]. However, a higher PVA concentration (40%) limited the formation of a porous structure, as high PVA concentration increased the solution viscosity and formed a thicker shell at the outer particle layer. This interpretation was supported by the SEM image of MPD-4, which revealed a spherical particle with continuous outer surface, suggesting limited external porosity and reduced internal surface exposure. These findings suggest that PVA facilitated porous architecture at moderate concentrations (20–30%), but excessively high PVA content may result in shell-dominated particle morphologies that reduce accessible surface area and internal porosity.

### 3.4. Powder Density and Flowability

Powder density, porosity, and flowability are displayed in Table 2. The successful development of large porous particles is characterized by low bulk density (~0.1 g/cm^3^ or less), low tapped density (less than 0.4 g/cm^3^), and high geometric diameters (5–30 μm) [25,26]. These characteristics generally correspond to an aerodynamic diameter within the optimal range for inhalation. In this study, spray-dried mannitol with a poring agent (MA) and co-spray-dried mannitol with diclofenac sodium (MD) did not yield desirable large porous particles, as they exhibited bulk densities above 0.1 g/cm^3^ and comparatively lower porosity values. In contrast, incorporating PVA at concentrations above 20% significantly reduced powder density and markedly increased porosity. The formulation containing 30% PVA achieved the lowest bulk density (0.03 g/cm^3^) alongside the highest porosity (97.76%). A highly porous particle structure is essential for inhalation, as it facilitates efficient aerosolization and dispersion, enabling deposition in the deep lung regions [27].

In this work, powder flowability and cohesiveness were evaluated using the relationship between bulk and tapped density that were presented as Carr’s index values. The lower Carr’s index value indicated lower powder cohesiveness, which could contribute to improved dispersibility and aerosolization. The results revealed that co-spray drying with PVA significantly decreased Carr’s index values, which gradually decreased with the increase in PVA concentrations. These results suggest that co-spraying with PVA led to smoother particle surfaces and reduced moisture sorption, which may help decrease interparticle friction. Such improvements can enhance powder dispersibility and potentially support better aerosol performance, as suggested in previous studies [6]. Powder true density reflected intrinsic porosity within the particles. This result was highly correlated with the SSA values, as PVA played a crucial role in pore formation, as described earlier.

### 3.5. FT-IR Spectroscopy

**Figure 2:**
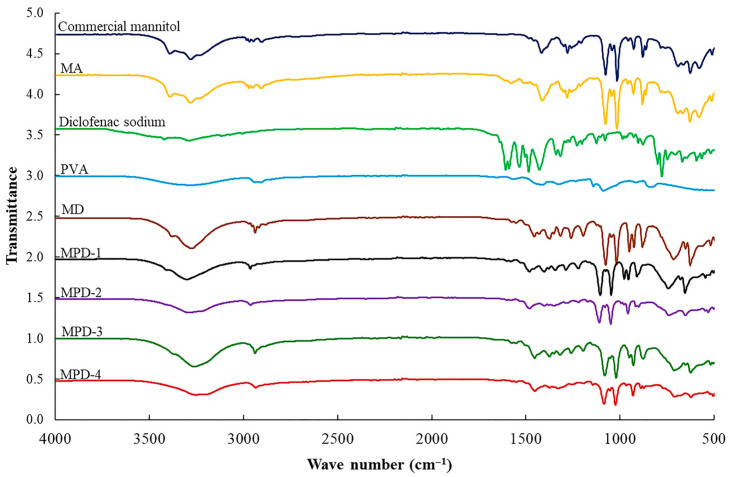
FTIR spectra of commercial mannitol, diclofenac sodium, PVA, MA, MD, and MPDs.

The FT-IR spectra of the samples are presented in Figure 2. The FT-IR spectra for commercial mannitol and MA revealed their correspondence to the β polymorph, as previously reported in our work [7]. The difference in polymorphism was characterized by the broad peak of O-H stretching between 3200 and 3600 cm^−1^, a C-H stretching peak at 2800–3000 cm^−1^, and C-H and C-O stretching vibration peaks between 1000 and 1400 cm^−1^. Notably, the single peak at 881 cm^−1^ in the fingerprint region further confirmed the presence of the β polymorph. [28] Thus, the spray drying process, as well as co-spray drying with ammonium bicarbonate, did not influence the polymorphism of mannitol. The spectrum of diclofenac sodium revealed a prominent peak at 1575 cm^−1^ corresponding to the carboxylate group (COO^−^), while a peak at 1600 cm^−1^ signified the aromatic C=C stretching vibrations. In addition, peaks at 3251 cm^−1^ and 3387 cm^−1^ were attributed to N-H stretching vibrations, consistent with previous reports [29,30]. PVA spectra showed a broad peak around 3200–3500 cm^−1^, attributed to the O-H stretching vibrations, while peaks in the region 2900–3000 cm^−1^ correspond to C-H stretching vibrations. The O-H bending vibration of the hydroxyl group was observed at 1413 cm^−1^, whereas 1560 cm^−1^ corresponded to C=C stretching. Additionally, C-O stretching and C-C stretching peaks were observed at 1087 and 838 cm^−1^, respectively, consistent with previous reports [31].

For the co-processed materials (MD and MPDs), the co-spray drying with diclofenac sodium caused changes in the mannitol polymorph to the predominate α form. The FTIR spectra of MD showed a broader peak of O-H stretching between 3200 and 3600 cm^−1^ compared to the original mannitol and MA, while the C-H and C-O stretching vibration peaks between 1000 and 1400 cm^−1^ corresponded to the α polymorph. For the MPD samples, MPD-1 exhibited an FTIR spectrum similar to that of the MD formulation, indicating the predominance of the α polymorph, as observed in the fingerprint region (400–1500 cm^−1^). However, increasing concentrations of PVA (MPD-2 to MPD-4) resulted in FTIR spectra characteristic of mixed α and δ polymorphs, evident in the fingerprint region. This FTIR observation was in agreement with XRD and DSC results showing that PVA incorporation altered mannitol polymorphism. These results suggest that diclofenac sodium and PVA competed with mannitol for available water molecules, thereby disrupting the intermolecular hydrogen-bonded O-H network necessary to stabilize the β polymorph [32,33]. The transformation of the β polymorph, the most stable form, into a mixture of α and δ polymorphs has been reported to increase particle surface energy, which may contribute to improved powder deagglomeration and dispersion upon inhalation [34].

### 3.6. X-Ray Diffraction (XRD)

**Figure 3:**
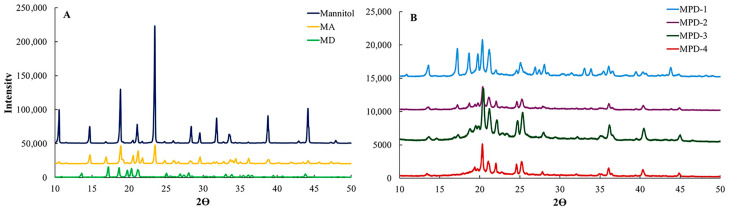
XRD patterns of (**A**) commercial mannitol, MA, and MD; and (**B**) MPDs.

The XRD patterns are shown in Figure 3. The XRD pattern of commercial mannitol revealed strong diffraction peaks at 10.5°, 14.6°, 18.8°, and 23.5°, which are characteristic of the β polymorph (Figure 3A) [34]. The co-spray drying with ammonium bicarbonate (MA) also provided an identical XRD pattern to the commercial mannitol, indicating that the ammonium bicarbonate and spray drying condition did not cause changes in the mannitol polymorph as reported in our previous work. On the other hand, the incorporation of diclofenac sodium and/or PVA significantly altered the mannitol polymorph. The MD which co-spray drying with diclofenac sodium showed strong peaks at 13.6, 17.1, 18.6, 20.3, and 21.2°, which correlated well with the α polymorph. Typically, diclofenac sodium has been reported to show peaks at 11, 15, 20, 23, 27, and 28°, which were not observed in the XRD patterns of MD and MPD. This result suggested the diclofenac sodium present in the co-spray drying particles was in an amorphous state, which favored drug dispersion in the matrix [35].

The MPD formulations (MPD-1 to MPD-4) showed a more pronounced reduction in diffraction peak intensity compared to MA and MD (Figure 3B), indicating a progressive decrease in crystallinity as a result of co-spray drying with PVA. A broad characteristic peak was observed between 18 and 24° 2θ, corresponding to the XRD pattern of PVA [36]. MPD-1 displayed a mixed XRD pattern of α- and β polymorphs of mannitol. In contrast, MPD-2 to MPD-4 were dominated by the α polymorph, with the emergence of δ polymorph peaks at 19.4°, 22.1°, 24.7°, 36.0°, and 40.3°, which became increasingly prominent at higher PVA concentrations, particularly in MPD-3 and MPD-4. The influence of PVA on the mannitol polymorphism was corroborated by FTIR and DSC analyses, both of which confirmed the transformation to a mixed α/δ polymorphic composition.

### 3.7. Differential Scanning Calorimetry (DSC)

**Figure 4:**
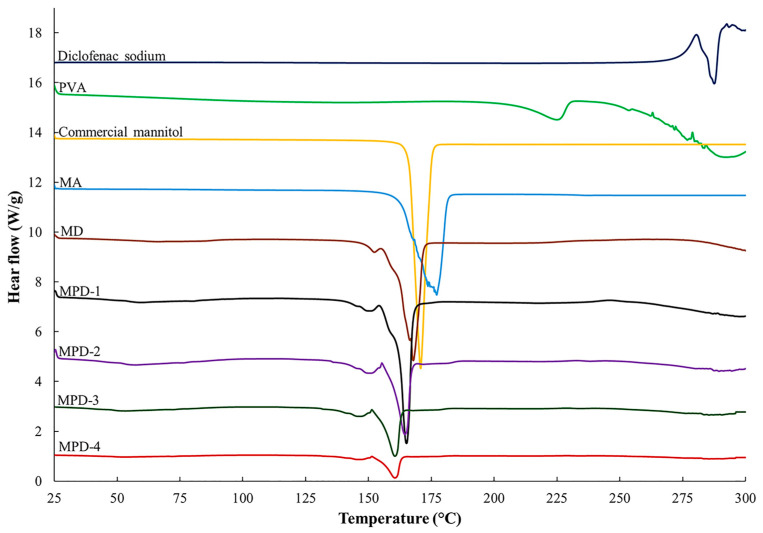
DSC thermograms of commercial mannitol, diclofenac sodium, PVA, MA, MD, and MPDs.

Thermal properties of the samples are illustrated in Figure 4 and Table 3. The commercial mannitol exhibited an endothermic melting peak at 166 °C, which corresponded to the β polymorph when co-interpreted with the XRD and FTIR results [37]. The co-spray drying with ammonium bicarbonate caused a shift and broadening of the mannitol melting peak, observed at 162 °C as reported in our previous work. For MD particles, the mannitol melting peak was observed at 161 °C, suggesting that the δ form of mannitol did not predominate in MD particles. The β and α polymorphs of mannitol exhibited similar melting points (166.5 °C and 166.0 °C, respectively), while the δ polymorph had a lower melting point (155 °C) [34]. The DSC thermogram of diclofenac sodium showed an endothermic shape peak at 283 °C that represented the melting peak. However, the melting peak of diclofenac sodium was absent in the DSC thermogram of MD and MPD, which indicated the amorphous state of diclofenac sodium, consistent with previous findings [35,38].

In the case of MPD particles, progressive shifts in thermal transitions were observed with an increase in PVA concentrations. MPD-1 showed a melting peak at 161 °C, which was close to MA and MD particles. However, a consistent reduction in melting point and enthalpy was recorded as PVA concentration increased, indicating a disruption of mannitol crystallinity and increased amorphous content due to PVA incorporation. Additionally, MPD-3 and MPD-4 exhibited endothermic onsets around 155 °C, corresponding to the δ polymorph of mannitol and consistent with FTIR and XRD findings, which confirmed that PVA incorporation altered mannitol polymorphism. The pure PVA showed a melting peak at 208 °C; however, this peak was absent in the MPD thermograms, indicating a loss of PVA crystallinity in the MPD particles [39].

### 3.8. In Vitro Drug Release Testing and Drug Content Percentage

**Figure 5:**
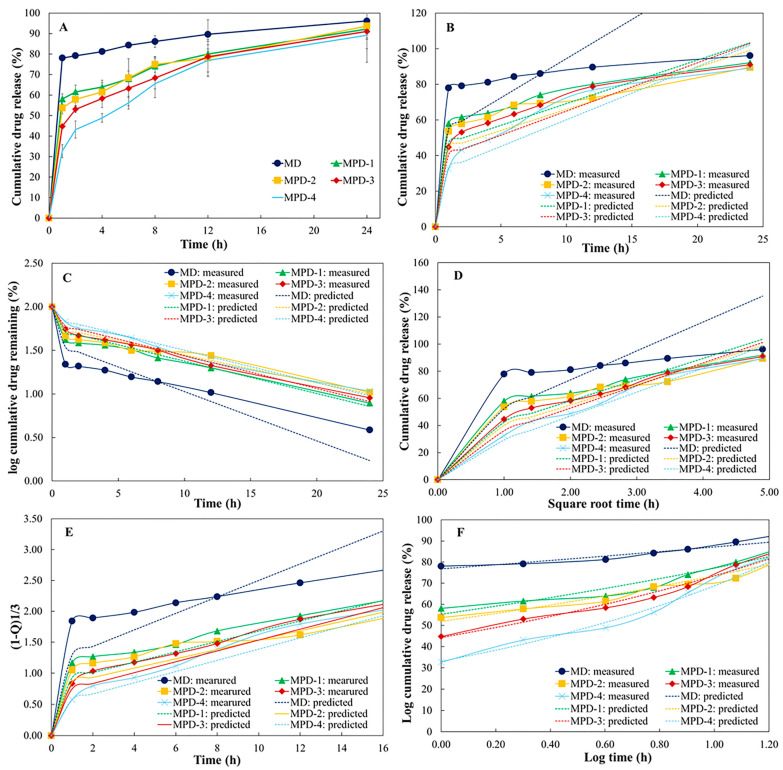
Cumulative drug release profiles (**A**) and release kinetics of MD and MPD formulations fitted to zero-order (**B**), first-order (**C**), Higuchi (**D**), Hixson–Crowell (**E**), and Korsmeyer–Peppas (**F**) models.

The cumulative drug release (%) and drug content percentage are illustrated in Figure 5 and Table 4. The drug content percentage study revealed that the MPD formulations exhibited a variation in drug content within ±5%, while the MD formulation yielded the lowest drug content percentage with over 5% variation. The presence of PVA appeared to enhance drug loading capacity by forming a cohesive matrix that promoted drug embedding within the particles, consistent with previously reported mechanisms [40]. For the in vitro dissolution testing, the results revealed that almost 80% of diclofenac sodium was released from the MD particle within the first hour of study, implying that the drug release from the particle could not be extended. In contrast, the co-spray drying with PVA significantly prolonged the drug release from the MPD particles, as PVA formed a hydrated, gel-like matrix upon contact with the dissolution media that became a barrier for drug diffusion. Therefore, diclofenac sodium was likely released from the particles via diffusion and matrix erosion processes, as influenced by the surrounding medium [22]. An increase in PVA concentration led to a corresponding decrease in drug release rate. MPD-4, which contained the highest PVA content, exhibited the most prolonged release profile due to PVA-restricted drug diffusion.

Release kinetics were evaluated using various mathematical models (Table 3). Among these, the Korsmeyer–Peppas model demonstrated the best fit across all MPD formulations, with the highest correlation coefficients (R^2^ = 0.9286–0.9861) and a low residual sum of squares (RSS = 22.16–57.20). The calculated *n* values for the MPD formulations ranged from 0.90 to 0.98, which are characteristic of super case II transport. This implies that drug release was controlled by a combination of diffusion and polymer chain relaxation or swelling mechanisms [41], consistent with previously reported behavior of PVA-based controlled release systems [42]. Additionally, all MPD formulations also showed a good fit to the first-order model, followed by the Higuchi model, indicating that drug release from the mannitol-PVA microparticles involved both a concentration-dependent mechanism and diffusion-controlled release from the polymer matrix [43,44]. In contrast, the zero-order and Hixson–Crowell models showed lower correlation (R^2^ < 0.75 in most cases), suggesting they were less suitable for describing the release behavior in this system.

However, the burst release was observed in all formulations during the first hour of dissolution testing (33–58%) and gradually reduced with the increase in PVA concentration. This effect could result from the presence of mannitol, as it was highly dissolved in the dissolution media that allowed the exposure of diclofenac sodium that embedded near the particle surface. After the initial burst release in the first hour, the cumulative release profiles showed extended release over a period of time. The burst release followed by extended release could be beneficial for specific purposes such as in anti-inflammatories, as it would provide an initial burst providing rapid relief, followed by extended release to support gradual healing over time [45].

## 4. Conclusions

In this study, large porous mannitol-PVA microparticles (MPDs) were successfully developed via spray drying for extended drug release. The incorporation of PVA significantly influenced the morphology, porosity, and drug release behavior of the spray-dried particles. PVA co-spray drying resulted in larger, more spherical particles with a smooth surface, enhancing flowability and aerosol performance. The large geometric diameter (>5 μm) and low bulk density (<0.1 g/cm^3^) are characteristic physicochemical properties typically associated with inhalable particles. Pore formation was facilitated by PVA and ammonium bicarbonate, contributing to low-density particles with a high specific surface area (SSA). Higher PVA concentrations reduced interparticle friction, leading to low Carr’s Index values and improved powder dispersibility and aerosolization efficiency. XRD, DSC, and FTIR analyses collectively confirmed that PVA altered the polymorphic form of mannitol from the β-dominant phase to a mixture of α and δ polymorphs as the PVA concentration increased. This transition is likely to enhance surface energy, thereby promoting powder dispersibility upon inhalation. The polymeric network formed by PVA critically modulated drug release, as its gel-forming properties created a diffusion barrier, thereby prolonging release from the matrix system. The formulation containing 40% PVA (MPD-4) exhibited the optimal extended-release profile, following first-order and Higuchi kinetics, indicating a diffusion-driven mechanism facilitated by the porous architecture. While an initial burst release (~30%) was observed, it was followed by extended release, which could be advantageous for therapies requiring an immediate therapeutic effect followed by prolonged drug action. In conclusion, the MPD-4 formulation demonstrated promising characteristics for extended-release drug delivery with physicochemical attributes commonly associated with inhalable microparticles. However, aerosol performance and in vivo lung deposition were not evaluated in this work. Future work should incorporate cascade impactor analysis and in vivo studies to confirm the aerodynamic performance and lung-deposition potential of these particles.

## Figures and Tables

**Table 1:** Co-spray drying conditions, particle size, and aerodynamic diameter of the samples.

Samples	Mannitol (%)	PVA (%)	Diclofenac Sodium (%)	Particle Size					Aerodynamic Diameter (μm)
				**D_10_ (μm)**	**D_50_ (μm)**	**D_90_ (μm)**	**Average** **Size (μm)**	**Span**	
MA	100	-	-	5.86 ± 0.25 ^b^	9.36 ± 0.32 ^b^	13.52 ± 0.52 ^a^	9.26 ± 0.29 ^a^	0.82	5.07
MD	90	-	10	4.94 ± 0.16 ^a^	8.54 ± 0.29 ^a^	11.89 ± 0.59 ^a^	8.63 ± 0.24 ^a^	0.81	4.40
MPD-1	80	10	10	7.54 ± 0.04 ^c^	13.36 ± 0.09 ^c^	23.47 ± 0.36 ^b^	13.74 ± 0.10 ^b^	1.19	6.27
MPD-2	70	20	10	8.58 ± 0.09 ^d^	14.94 ± 0.14 ^d^	25.29 ± 0.14 ^b^	15.48 ± 0.35 ^c^	1.12	3.79
MPD-3	60	30	10	9.40 ± 0.17 ^e^	15.49 ± 0.32 ^d^	24.49 ± 0.82 ^b^	15.83 ± 0.19 ^c^	0.97	3.17
MPD-4	50	40	10	9.24 ± 0.07 ^e^	18.37 ± 0.15 ^e^	35.27 ± 0.91 ^c^	18.38 ± 0.13 ^d^	1.42	4.50

A common letter (a–e) is not significantly different within group by Tukey HSD test at the 5% level of significance (*p* < 0.05). Shape factors were assigned as follows: MA, MPD-2, MPD-3, MPD-4 = 1.0; MPD-1 = 1.2; and MD = 1.5.

**Table 2:** Powder densities, specific surface area, and powder flowability of the samples.

Samples	Bulk Density (g/cm^3^)	Tapped Density (g/cm^3^)	True Density (g/cm^3^)	Porosity (%)	SSA (m^2^/g)	Carr’s Index (%)
MA	0.18 ± 0.01 ^d^	0.29 ± 0.01 ^d^	1.3849 ± 0.0144	87.00	4.356	39.43 ± 0.56 ^d^
MD	0.22 ± 0.01 ^e^	0.38 ± 0.01 ^e^	1.4899 ± 0.0079	85.23	3.266	41.29 ± 1.12 ^de^
PMD-1	0.14 ± 0.00 ^c^	0.25 ± 0.00 ^c^	1.5309 ± 0.0129	90.85	4.863	43.94 ± 1.06 ^e^
PMD-2	0.04 ± 0.00 ^ab^	0.06 ± 0.00 ^b^	1.4459 ± 0.0236	97.23	5.823	36.01 ± 0.62 ^c^
PMD-3	0.03 ± 0.00 ^a^	0.04 ± 0.00 ^a^	1.3389 ± 0.0373	97.76	7.404	27.46 ± 1.37 ^b^
PMD-4	0.05 ± 0.00 ^b^	0.06 ± 0.00 ^ab^	1.5839 ± 0.0217	96.84	4.388	17.56 ± 1.55 ^a^

A common letter (a–e) is not significantly different within group by Tukey HSD test at the 5% level of significance (*p* < 0.05).

**Table 3:** Thermal properties of the samples.

Sample	T_oneset_ (°C)	T_peak_ (°C)	T_end_ (°C)	Enthalpy (J/g)
Diclofenac sodium	283.33	287.50	289.81	64.88
PVA	208.26	225.00	229.98	67.84
Commercial mannitol	166.02	170.83	174.60	278.54
MA	161.67	177.17	181.38	274.31
MD	160.67	167.83	171.83	231.90
MPD-1	161.33	165.17	167.72	213.64
MPD-2	158.73	164.50	167.07	108.84
MPD-3	154.98	160.67	164.80	81.75
MPD-4	155.33	160.83	163.87	54.87

**Table 4:** Release kinetics of MD and MPD formulations.

Formulations	Drug Content (%)		
MD	91.35 ± 0.99		
MPD-1	104.64 ± 1.30		
MPD-2	98.91 ± 1.22		
MPD-3	95.08 ± 0.42		
MPD-4	97.45 ± 0.31		
Formulations	**Zero-order**		
	Correlation Coefficient	Residual Sum of Squares	
MD	0.3521	1348.17	
MPD-1	0.4921	673.35	
MPD-2	0.5059	600.92	
MPD-3	0.6139	578.54	
MPD-4	0.7243	564.38	
Formulations	**First-order**		
	Correlation Coefficient	Residual Sum of Squares	
MD	0.5821	281.25	
MPD-1	0.8776	199.09	
MPD-2	0.8505	216.56	
MPD-3	0.9308	121.58	
MPD-4	0.9576	68.79	
Formulations	**Higuchi**		
	Correlation Coefficient	Residual Sum of Squares	
MD	0.5494	1291.84	
MPD-1	0.7535	576.95	
MPD-2	0.7638	508.52	
MPD-3	0.8655	317.25	
MPD-4	0.9428	169.17	
Formulations	**Hixson–Crowell**		
	Correlation Coefficient	Residual Sum of Squares	
MD	0.4861	478.42	
MPD-1	0.7607	250.63	
MPD-2	0.7462	268.30	
MPD-3	0.8457	176.07	
MPD-4	0.8985	813.13	
Formulations	**Korsmeyer–Peppas**		
	Correlation Coefficient	Residual Sum of Squares	*n* Value
MD	0.9291	7.47	0.9017
MPD-1	0.9286	57.20	0.9286
MPD-2	0.9434	54.71	0.9434
MPD-3	0.9854	22.16	0.9854
MPD-4	0.9861	36.21	0.9861

## Data Availability

The original contributions presented in this study are included in the article. Further inquiries can be directed to the corresponding author.

## Abbreviations

The following abbreviations are used in this manuscript:

AB	Ammonium bicarbonate
MA	Spray-dried mannitol with AB
MD	Co-spray-dried mannitol–diclofenac sodium
MPDs	Co-spray-dried PVA-mannitol, diclofenac sodium, and AB
